# The osteoblast secretome in *Staphylococcus aureus* osteomyelitis

**DOI:** 10.3389/fimmu.2022.1048505

**Published:** 2022-11-22

**Authors:** Valentina Granata, Valentina Possetti, Raffaella Parente, Barbara Bottazzi, Antonio Inforzato, Cristina Sobacchi

**Affiliations:** ^1^ IRCCS Humanitas Research Hospital, Rozzano, Italy; ^2^ Milan Unit, National Research Council - Institute for Genetic and Biomedical Research (CNR-IRGB), Milan, Italy; ^3^ Department of Biomedical Sciences, Humanitas University, Pieve Emanuele, Italy

**Keywords:** osteomyelitis, infection, bone, osteoblast, staphylococcus aureus, cytokines, chemokines

## Abstract

Osteomyelitis (OM) is an infectious disease of the bone predominantly caused by the opportunistic bacterium *Staphylococcus aureus* (*S. aureus*). Typically established upon hematogenous spread of the pathogen to the musculoskeletal system or contamination of the bone after fracture or surgery, osteomyelitis has a complex pathogenesis with a critical involvement of both osteal and immune components. Colonization of the bone by *S. aureus* is traditionally proposed to induce functional inhibition and/or apoptosis of osteoblasts, alteration of the RANKL/OPG ratio in the bone microenvironment and activation of osteoclasts; all together, these events locally subvert tissue homeostasis causing pathological bone loss. However, this paradigm has been challenged in recent years, in fact osteoblasts are emerging as active players in the induction and orientation of the immune reaction that mounts in the bone during an infection. The interaction with immune cells has been mostly ascribed to osteoblast-derived soluble mediators that add on and synergize with those contributed by professional immune cells. In this respect, several preclinical and clinical observations indicate that osteomyelitis is accompanied by alterations in the local and (sometimes) systemic levels of both pro-inflammatory (e.g., IL-6, IL-1α, TNF-α, IL-1β) and anti-inflammatory (e.g., TGF-β1) cytokines. Here we revisit the role of osteoblasts in bacterial OM, with a focus on their secretome and its crosstalk with cellular and molecular components of the bone microenvironment and immune system.

## Introduction

Osteomyelitis is a severe bone infection arising from hematogenous spread of pathogens, mainly in pediatric patients (1), or direct contamination of the bone after fracture or surgery, more commonly in adults (2). Typically associated with comorbidities, osteomyelitis has a relatively high incidence amongst diabetics, where it develops secondary to vascular and neuropathic complications of hyperglycemia, and may require extreme clinical measures (i.e., limb amputation) (3). Osteomyelitis occurs also in in the absence of known risk factors for invasive infections, which underlines the complexity of its etiopathogenesis. Several pathogens, including bacteria, fungi, and viruses, can cause bone infections, but the most common etiologic agent of the disease is the Gram-positive bacterium *Staphylococcus aureus* (*S. aureus*), which is responsible for up to 60% of the cases (4). Different classification schemes have been proposed for osteomyelitis, based on a range of clinical and microbiological characteristics (5). Most commonly, the disease is classified as either acute or chronic, according to its duration, and either hematogenous or contiguous, according to the origin of infection. During a bone infection, the serum levels of several cytokines [e.g., IL-6, IL-8, IL-1β, IL-12(p70)], angiogenic factors (e.g., VEGF), and acute phase proteins (e.g., C reactive protein, CRP) increase, which is of diagnostic value in the clinical handling of the disease; nevertheless, specific infection markers with diagnostic value are still sought for (6, 7).

The first line of defense against invading pathogens, including *S. aureus*, is represented by cells of the innate immune system (neutrophils, monocytes, macrophages), which detect the pathogens through germline-encoded pattern-recognition receptors (PRRs), including Toll-like receptors (TLRs) and cytoplasmic receptors. PRRs recognize specific microbial components known as pathogen-associated molecular patterns (PAMPs), such as LPS, lipoteichoic acid, lipoproteins, and peptidoglycans, and convey the biochemical signals that are ultimately responsible for activation of the immune cells (8). PRRs are also made by non-immune cells, including cells of the osteoclast and osteoblast lineage in the bone. All TLRs but TLR2 and TLR4 are downregulated during osteoclast differentiation, and relative timing of TLR and RANKL/MCSF stimulation of osteoclast precursors elicit opposite effect on osteoclast formation (9). Very recently it has been reported that TLR2 and TLR9 signaling contributes marginally to the inflammatory bone loss and enhanced osteoclast formation that accompany *S. aureus*-dependent osteomyelitis (see *Osteoclasts and their progenitors*) (10). However, it is generally accepted that osteoblasts use TLRs and NODs to recognize and respond to *S. aureus*, which leads to secretion of the master osteoclastogenic cytokine RANKL as well as other factors (11), as discussed below.

**Figure 1:**
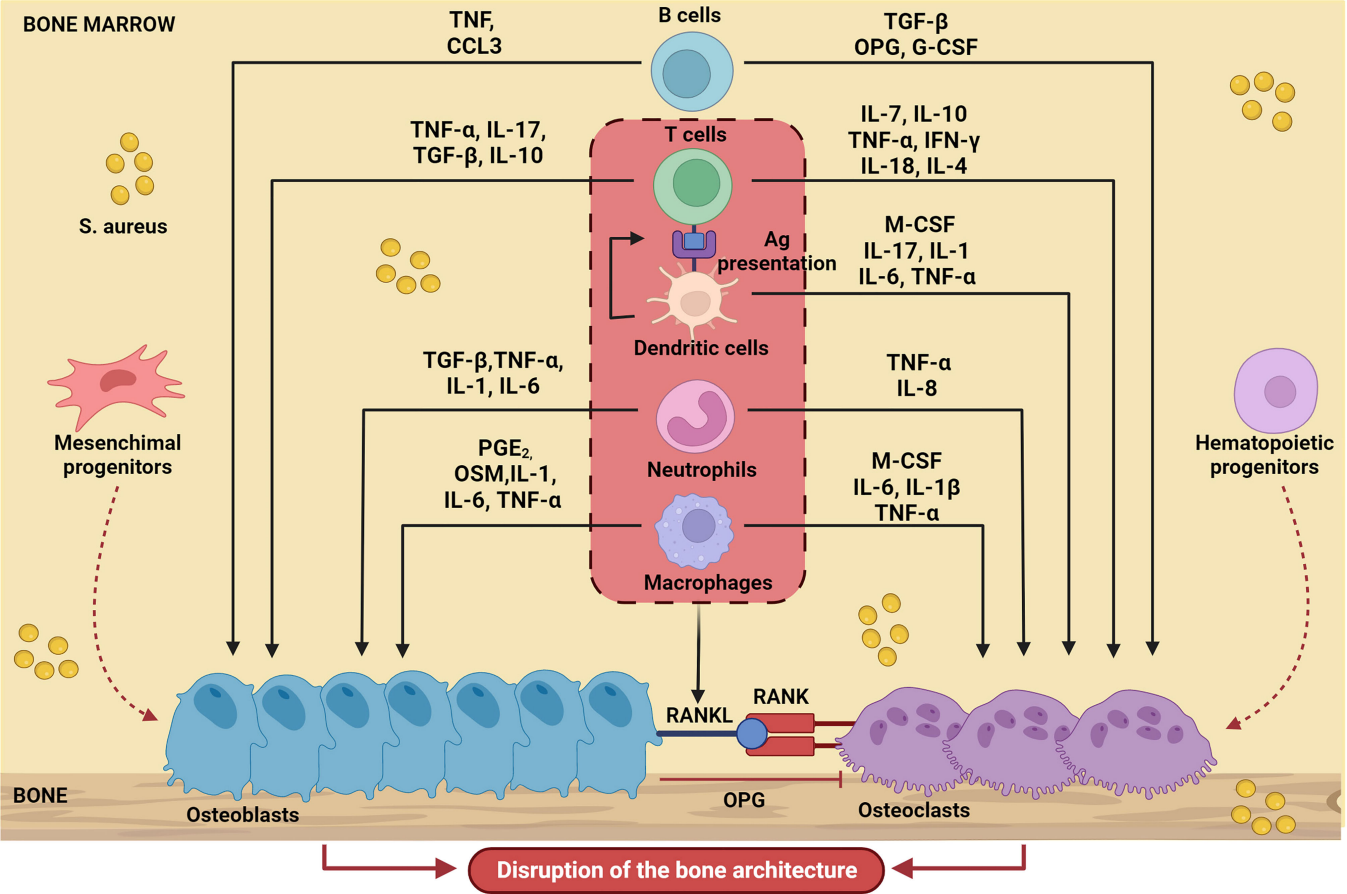
Schematic representation of bone cell regulation by the main soluble factors produced by immune cells during S. aureus-induced osteomyelitis. Upon infection, immune cells migrate into the bone microenvironment and release cytokines (IL-17, IL-10, IL-1, IL-1β, IL-6, IL-7, IL-18, IL-4, IL-8), chemokines (CCL3), growth factors (M-CSF, G-CSF) and other several inflammatory mediators (TNF-α, PGE_2_, TGFβ, INF-γ, OSM) that influence osteoblast and osteoclast activity. The result is massive disruption of the normal bone architecture. The Figure was created with BioRender.com.

In line with the general paradigm of immunological activation upon PRR engagement, in the context of a bone infection, polymorphonuclear leukocytes (PMNs) and macrophages migrate into the bone microenvironment and therein release several diverse inflammatory mediators, including cytokines, chemokines (e.g., IL-1β, IL-6, and TNF-α, CCL3 and CXCL2), and other factors. The immune reaction that mounts in the bone alters the local homeostasis profoundly, which adds on the direct effects of *S. aureus* on skeletal cells (8). Proinflammatory mediators promote formation and activation of osteoclasts, thus enhancing bone resorption; in parallel, new, poorly structured bone is deposited at the periosteum (periosteal bone reaction) by osteoblasts to confine the infection. These opposing processes collectively result into progressive disruption of the bone microarchitecture. The crosstalk between immune and skeletal cells in the context of bone infections sustained by *S. aureus* (a paradigmatic example of osteoimmunology) is schematically depicted in **Figure 1**.

**Figure 2:**
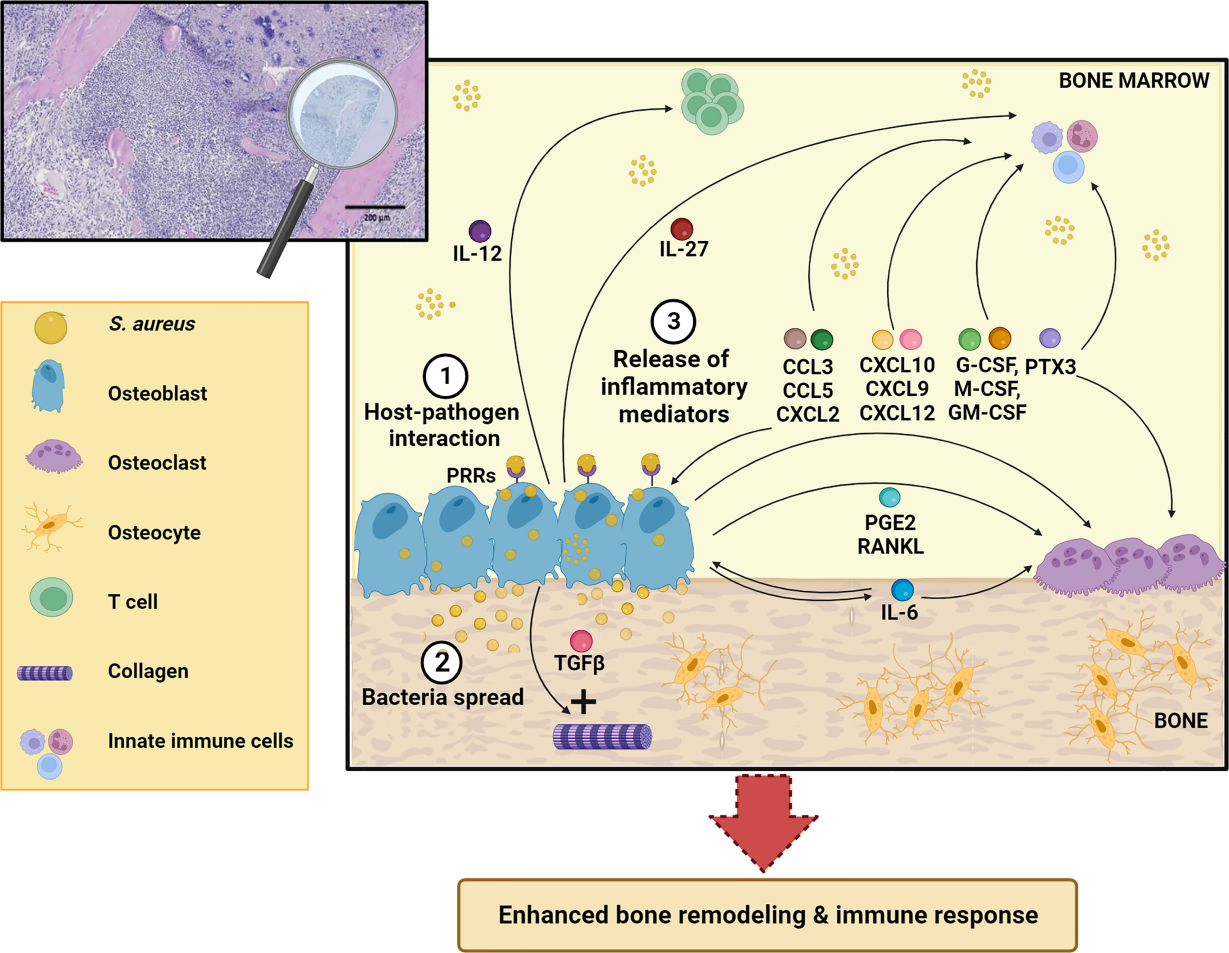
Schematic representation of osteoblast response to *S. aureus* infection. After osteoblast infection (1), *S. aureus* can grow and spread further in the bone (2) and induce synthesis and release of different inflammatory mediators (IL-12, IL-27, IL-6, CCL3, CCL5, CXCL2, CXCL10, CXCL9, CXCL12, TGFβ, G-CSF, M-CSF, GM-CSF, PTX3, RANKL, PGE_2_) (3) that act both on skeletal and immune cells. This leads to enhanced bone remodeling and immune response. See the main text for more details. The Figure was created with BioRender.com.

Besides their function in bone homeostasis, both osteoclasts and osteoblasts exert immune regulatory functions by orchestrating synthesis and release of various inflammatory molecules (12). In the present review, we present and discuss the roles of osteoblasts in the pathogenesis of osteomyelitis, with a focus on osteoblast-derived soluble factors and their contribution to the long-term fate of bone infections (**Figure 2**).

## Mainstays in bone biology and basic microbiology of *S. aureus* osteomyelitis

Bone is a dynamic tissue that undergoes constant remodeling throughout life (13). Osteoclasts and osteoblasts are the two cell types primarily involved in bone remodeling, responsible for bone resorption and bone formation, respectively. Osteoclasts are of hematopoietic origin, and unique in their ability to degrade the inorganic (hydroxyapatite) and organic (mostly collagen) matrix of the bone (14) through acidification of the resorption lacuna and release of hydrolytic enzymes (e.g., cathepsin K). Conversely, osteoblasts arise from cells of the mesenchymal lineage, and produce the whole set of components of the mineralized extracellular matrix of the bone, which comprises collagen, proteoglycans and several non-collagenous proteins, and an extremely dense hydroxyapatite-based mineral (15). As the matrix grows, some osteoblasts are enclosed in it and become osteocytes. These are terminally differentiated cells that communicate with each other and other cell types by means of cellular processes that penetrate the canaliculi in the bone extracellular matrix.

The crosstalk between osteoclasts and osteoblasts shapes the extracellular matrix. Their mutual interaction relies on a series of membrane-bound molecules and soluble factors (e.g., EFNB2-EPHB4, FASL-FAS and SEMA3A-NRP1). Among the latter, M-CSF and RANKL, produced by osteoblasts and acting on their specific receptors on the osteoclast surface (C-FMS and RANK, respectively), have a pivotal role in bone homeostasis (14). In addition, osteoblasts release OPG, the decoy receptor for RANKL that hinders RANK-RANKL interaction, reducing osteoclast formation and bone resorption. In fact, RANKL/OPG ratio is commonly used as an indicator of the balance between bone formation and resorption (16).

*S. aureus* can invade, colonize, and thrive in the bone. Microbial surface components recognizing adhesive matrix molecules (MSCRAMMs), such as fibronectin-binding protein A and B (FnBPA and B), collagen adhesin (Cna) and Staphylococcus protein A (SpA) (17), initiate adhesion to the bone extracellular matrix. Once bound to the matrix, *S. aureus* can activate diverse mechanisms to escape the host immune response, survive in the bone microenvironment, and establish chronic infection. A primary strategy of immune evasion is biofilm formation, a complex process that involves synthesis and release of extracellular polymeric substances (i.e., polysaccharides and extracellular DNA) and is controlled both by microbial (i.e., the Agr quorum-sensing system) (18) and host factors (i.e., mineral components of the bone matrix, such as Mg^2+^ and Ca^2+^, oxygen and nutrient availability) (18). Biofilms reduce osteoblast viability and increase RANKL production, thus promoting bone resorption (19). Moreover, in an *in vitro* dynamic model of biofilm deposition, the supernatant of TNFα-treated osteoblasts was found to affect *S. aureus* adhesion and biofilm formation (20). Abscess formation is another survival strategy for *S. aureus*, whereby following upon the engagement of fibrinogen by the bacterial clamping factors A and B (ClfA and B) and its conversion to fibrin (by coagulase, CoA, and von Willebrand factor-binding protein, vWbp), a fibrous pseudocapsule is formed that encases a core of staphylococcal abscess communities (SACs) and is surrounded by a layer of necrotic leukocytes (mostly, neutrophils) (2). Furthermore, *S. aureus* is able to invade the osteocyte lacunocanalicular network through bacteria deformation (whereby a key role has been proposed for the transpeptidases penicillin-binding proteins 3 and 4), entry and migration into the canaliculi *via* asymmetric binary fission at the leading edge (21, 22); these mechanisms are particularly relevant to long term bacterial persistence and eradication. Finally, intracellular infection and survival is a bacterial strategy to evade immune surveillance; of note, all skeletal cells are potential targets, as discussed in the following section.

## Skeletal cell infection in the context of *S. aureus* osteomyelitis

### Osteoclasts and their progenitors

A growing body of evidence supports the concept of osteoclasts working as the innate immune cells of the bone (23, 24); accordingly, it is expected that they play a role in the framework of osteomyelitis. Bacterial infection has the potential to influence osteoclast formation directly, through receptor ligation on progenitor cells (9), and indirectly, through enhanced cytokine release from neighboring cells (8). In particular, *in vitro S. aureus* infection of murine bone marrow-derived osteoclast precursors has been shown to induce their differentiation into activated macrophages that actively secrete proinflammatory cytokines, such as CCL5, MIP-1α, MIP-1β, G-CSF, IL-12p40, and MCP-1 (25). These cytokines enhance the bone resorption capacity of uninfected mature osteoclasts and differentiation of uninfected precursors. Moreover, infection of mature osteoclasts directly promotes cell fusion, which contributes to enhance their ability to resorb bone (25). A similar effect has been also demonstrated upon exposure of human monocyte-derived osteoclasts to Staphylococcal superantigen TSST-1, while the Panton-Valentine leukocidin, known as one of the most powerful pore-forming toxins, and hemolysin-α induced osteoclast death, indicating that the clinical presentation and outcome of bone and joint infection could be related, at least partly, to the toxin profile of the *S. aureus* isolate involved (26). The osteoclast intracellular infection, which has been demonstrated also *in vivo* (27), has been recently studied exploiting fluorescently labelled bacterial strains visualized by confocal and time-lapse microscopy (28). Intracellular penetration of bacteria occurred *in vitro* in a short timeframe; bacterial proliferation started two-four hours post-infection, but the bacterial load in the intracellular compartment of infected osteoclasts did not change over time (28). Osteoclast colonization was accompanied by reduced bactericidal potential compared to non-infected osteoclast precursors (29). Moreover, the proliferative capacity of *S. aureus* within osteoclasts was dependent on the signaling cascade activated by the osteoclastogenic master transcription factor NFATc1, and varied to some extent amongst individual osteoclasts (29).

### Osteoblasts and their progeny

*S. aureus* has been shown to infect osteoblasts and downstream mature cells (osteocytes) both in *in vitro* and *in vivo* animal models and clinical biopsies (21, 30–33). The interaction of osteoblasts with *S. aureus* involves several pathogen-derived factors and components of the osteoblast plasma membrane. For example, a major virulence factor of *S. aureus*, SpA, interacts directly with osteoblasts *via* TNFR1, and activates intracellular signaling cascades that result into reduced proliferation, enhanced apoptosis, and impaired mineralizing potential of cultured osteoblasts (34). The binding of *S. aureus* FnBPA and FnBPB to the extracellular matrix protein fibronectin is recognized as another important process in osteoblast infection. Indeed, fibronectin is believed to act as a “molecular bridge” in linking osteoblasts to the pathogen through α5β1 integrin (35). In this respect, the supramolecular structure of fibronectin in the bone extracellular matrix has been recently shown to play an essential role in bacterial uptake by osteoblasts (36).

Whether bacteria internalization in osteoblasts is achieved passively or through an active process has been debated. It is likely that in fact both mechanisms are in place (37, 38). Intracellular infection has been proposed to occur very rapidly upon exposure to *S. aureus*. Indeed, bacteria have been detected by immunofluorescence microscopy inside murine calvaria osteoblast-like MC3T3-E1 cells as early as 15 minutes after infection, and the rate of bacteria internalization was found to increase over time (39). Invasion likely requires actin rearrangement at the cell surface; in fact, treatment with the actin depolymerization agent Cytochalasin D significantly reduced *S. aureus* invasion (40).

Mouton and colleagues recently demonstrated that *S. aureus* internalization (assessed *in vitro* through a gentamycin/lysostaphin protection assay that allows eliminating adherent and non-adherent bacteria, while sparing intracellular pathogen) induced an impairment in early osteoblast differentiation by interfering with β1 integrin signaling, leading to decreased expression of RUNX2 and COL1α1 and ALP activity. Accordingly, an internalization defective *S. aureus* strain lacking fnbpA expression did not elicit this effect (41). Consistent with this, *in vivo* infection with the *S. aureus* strain capable of cell internalization altered some bone histomorphometric parameters, supporting the hypothesis that osteoblast functions are impaired upon intracellular bacterial infection (41).

Osteoblast colonization by *S. aureus* stimulates the focal adhesion kinase (FAK)/epidermal growth factor receptor (EGFR) and c-Src signaling pathways by enhancing their phosphorylation in a time-dependent fashion; on the contrary, inhibition of the EGFR/FAK or c-Src signaling pathways significantly reduces the rate of pathogen internalization (39). Consequently, these pathways could be targeted in parallel to standard antibiotic therapy of chronic *S. aureus* osteomyelitis.

Osteoblast infection by *S. aureus* has been confirmed also in the presence of invasive MRSA infection of the human MG-63 osteosarcoma cell line, using imaging flow cytometry (IFC) which is more sensitive and reproducible than conventional cell culture methods (42). Pathogen uptake is known to vary depending on the strain irrespectively of antibiotic resistance (43). Statistical analysis of results obtained by IFC assays demonstrated that intracellular persistence capacity of several different MRSA strains over a 24 hour-timeframe was correlated with the total number of infected cells at 24 hour-post-infection and not with the number of bacteria that managed to enter/replicate in each single cell, defined by spot counting after cell transient permeabilization and pathogen staining with a membrane-impermeable green-fluorochrome vancomycin analogue (42). This would suggest that other factors besides the specific clone define the bacteria ability to internalize and persist inside osteoblasts. Future research is needed on this point that is relevant to chronicization of infection.

The encounter of the pathogen with osteoblasts results into release of cytokines and chemokines (as discussed later on) that recruit and activate immune cells; increased RANKL production that sustains osteoclast activity; impaired bone matrix production and mineralization; and ultimately osteoblast death through upregulation of the cell death signal transducer TRAIL and its cell surface death receptors, and concomitant decrease of the decoy receptor OPG (44, 45). All these events contribute to bone loss. Progression from acute to chronic bone and joint infections is accompanied by phenotypic adaptation of the pathogen to a less virulent form, called “small colony variant”. This is characterized by increased intracellular persistence and antibiotics resistance, and reduced cytokine release induction and immune system stimulation (46, 47). In particular, osteocytes have been recently demonstrated to constitute a reservoir for silent or persistent infection owing to reduced antimicrobial capacity to eliminate intracellular bacteria, and higher pathogen survival in the form of small colony variants (11, 48). Moreover, infected osteocytes can also elicit an inflammatory response that contributes to communication with other skeletal cells, immune cell recruitment and bone disruption (11).

The mechanisms described above are specific for *S. aureus* and not shared by other opportunistic bacteria involved in orthopedic infections, which points to the evolution of bacterial species-specific ways of interaction with eukaryotic cells that need to be further elucidated.

## Osteoblasts as inflammatory cells

### The osteoblast secretome in a non-infected setting

The secretory function is highly relevant in the framework of osteoblast activities. It comprises the release of the diverse components of the extracellular matrix, essentially calcium-deficient apatite and trace elements, for the inorganic part; type I collagen and other types of collagens, and non-collagenous proteins (γ-carboxyglutamate-containing proteins, proteoglycans, glycoproteins, and small integrin-binding ligands N-linked glycoproteins), for the organic part (49). Osteoblasts also release a variety of cytokines, chemokines, and growth factors (i.e., the osteoblast secretome) that regulate osteoclast formation and resorptive activity (e.g., M-CSF, RANKL, OPG, WNT5A, WNT16, IL-6, GM-CSF) (14), enhance the osteoblast anabolic function (e.g., IGFs and IGFBPs) (50), support hematopoiesis (e.g., G-CSF, osteopontin, thrombopoietin, angiopoietin 1, CXCL12, SCF and IL-7) (51) and act as immune modulators (e.g., IL-6, GM-CSF, CCL5) (52). In this respect, an intimate crosstalk takes place in the bone between skeletal cells and the immune system, which also involves the release of cytokines and chemokines in diverse pathophysiological contexts (53). Systemic and local factors such as inflammation and infection elicit marked changes in bone cell functions, as further described in the following sections.

### The osteoblast secretome in S. aureus osteomyelitis: Cytokines and growth factors

At sites of infection, soluble factors deriving from activated immune and skeletal cells make up an inflammatory milieu that mediates reciprocal regulation of the osteal and immune components and provides host defense against pathogens. In this regard, osteoblasts play an active role, and cannot be regarded as inert niches for bacterial colonization. In fact, not only do they strive to kill intracellular bacteria by increasing the production of reactive oxygen species and oxidative stress, but they also take part in the immune response orchestrated by professional innate immune cells through production of antimicrobial peptides (beta-defensins), as shown in *in vitro* and *in vivo* models and *ex vivo* in human specimens (54, 55). In addition, the osteoblast secretome further boosts the inflammatory response and affect the behavior of skeletal cells (see **Figure 2**).

Cytokine synthesis and release involve several intracellular signaling pathways including the NF-kB pathway, which regulates the secretion of IL-6 and MCP1 (CCL2; see below) (56), and the JNK pathway, which is activated downstream of *S. aureus* SpA binding to TNFR1 on the osteoblast plasma membrane and leads to increased TLR2 and RANKL protein levels and TNF-α and IL-6 secretion (56).

The transcriptomic profile of infected osteoblasts has been recently comprehensively investigated by RNAseq analysis of FACS-sorted *S. aureus*-bearing MG-63 cells, and compared to that of non-infected cells and of a mixed cell population comprising both infected and not-infected MG-63 cells (57). Specifically, this work indicated enhanced immune and inflammatory responses in a model of long-term infection, taking advantage of engineered bacterial strains expressing a fluorescent reporter gene that allowed isolation of infected cells 6 days after infection. Top up-regulated genes included several cytokines such as IL-33, IL-32, IL-6, IL-1β, IL-1α, IL-24, G-CSF, TRAIL and TNFSF14, with higher protein levels in culture supernatants. Accordingly, in the Gene Set Enrichment Analysis, several pathways enriched in FACS-sorted infected MG-63 were related to the immune response, including functional categories such as antigen processing and presentation (of note, CD44 and HLA-DR expression has been reported in cultured human osteoblasts) (58) complement and coagulation cascade, platelet activation, Th17 cell differentiation, IL-17 pathway, NOD-like and TLR signal cascades (known to be involved in bacteria recognition), and cytokine signaling (57).

At the protein level, IL-6 has been found to be upregulated in infected murine osteoblasts (in various experimental settings) and human bone tissues (59, 60). Increased IL-6 induces COX-2 and thereby PGE2 and RANKL, which modulate osteoclast recruitment and differentiation, contributing to progressive inflammatory damage and bone loss (61). Enhanced RANKL production by osteoblasts, without concomitant significant change in OPG expression, occurs also downstream of TLR2 recognition of *S. aureus*; accordingly, RANKL increase is abrogated in osteoblasts from Tlr2 knockout mice. This mechanism would support pronounced bone resorption and periosteal osteoclast formation in *S. aureus*-infected bones (62).

IL-12 also is secreted by osteoblasts in *S. aureus*-induced osteomyelitis (59). It has been proposed to strengthen Th1 immune responses and favor elimination of intracellular bacteria (63), a mechanism that could be potentially used to develop novel strategies for infection prevention (64, 65). Since IL-12 in the bone microenvironment promotes myeloid-derived suppressor cell recruitment, it is plausible that osteoblasts contribute to this mechanism (66).

Expression of the highly conserved anti-inflammatory cytokine TGFβ1 has been reported to change in MG-63 cells infected with four different clinically isolated *S. aureus* strains (43), with 2 of them causing downregulation in the short and one upregulation in the long timeframe (3 and 24 hours post infection, respectively). Among other functions, TGFβ1 stimulates type I collagen production (67), therefore an increase of this factor might explain the abnormal matrix deposition that occurs in the periosteum during an infection.

IL-1β and TNF-α increased in the bone of animals with *S. aureus*-induced OM (43, 68, 69), and both cytokines stimulated osteoclast maturation and function. However, some findings regarding to IL-1β are contradictory: *in vitro* infection of murine primary osteoblasts with *S. aureus* resulted in increased transcription, but not in increased protein synthesis or secretion. Of note, the same was observed for IL-18 (70), a potent inflammatory molecule, structurally and functionally closely related to IL-1β: in fact, it favors osteoclast differentiation by expanding the inflammatory response and inhibits the osteogenic function (71). Owing to the fine line between intense or exaggerated immune response, it is reasonable that IL-1β and IL-18 production is strictly controlled (70), however their regulation deserves further investigation. In parallel, the specific IL-18 inhibitor *IL-18BP* was upregulated *in vitro* in human primary osteoblasts 2 hours after *S. aureus* infection (https://www.ebi.ac.uk/arrayexpress/E-MTAB-6700), while no data are available regarding IL18-BP protein production by osteoblasts in this specific context, to the best of our knowledge. Therefore, at present, activated immune cells, not osteoblasts, are the most likely source of these ILs in the bone microenvironment during bone infections.

Also, while low levels of TNF-α were detected in *in vitro* differentiated human osteoblasts and in the MG-63 cell line in basal conditions (72), a marked increase was observed in MG-63 upon infection (43, 73). A recent study described a novel signaling cascade comprising TNF-α/miR-129-5p/endothelial nitric oxide synthase (eNOS) in the pathogenesis of osteomyelitis (74). Briefly, TNF-α and miR-129-5p were upregulated while eNOS was downregulated in *S. aureus*-infected MC3T3-E1 cells and in osteomyelitis patients’ blood. Accordingly, a TNF-α blocker inhibited miR-129-5p and elevated eNOS expression, likely contributing to rescue the mineralization defect caused by *S. aureus* infection in MC3T3-E1 cells (74).

IL-27 expression has been recently demonstrated to be induced early (on day 1) in the infected bone in a transtibial model of *S. aureus*-induced osteomyelitis and upon *in vitro* infection of MC3T3-E1 cells and primary osteoblasts (75). This cytokine likely contributes to host innate immune response in the early phases of the infection by stimulating local neutrophil recruitment and activation.

Finally, there is preliminary evidence of IFN-β secretion by mature murine osteoblasts in response to *S. aureus* infection (76). This type I interferon has been reported to be a negative regulator of RANKL-mediated osteoclastogenesis by inhibiting the translation of the critical signaling component c-Fos (77). Based on these data, *S. aureus* would stimulate osteoblasts to release factors with opposing effects on osteoclastogenesis (78); it can be speculated that the production of IFN-β represents a compensatory response to restore bone homeostasis.

Cytokine production from infected osteoblasts has been shown also in the framework of three-dimensional (3D) models of *S. aureus*-induced osteomyelitis, which more closely reproduce composition and structure of the natural bone compared to conventional 2D culture systems. For example, in a 3D model of osteomyelitis based on the coculture of MC3T3-E1 cells and *S. aureus* on magnesium-doped hydroxyapatite/collagen I scaffolds, *Tnf-α* expression increased over time during infection. The same was observed for the long pentraxin *Ptx3*, a key pattern recognition molecule with emerging roles in bone pathophysiology, known to be induced by TNF-α. On the contrary, *Tgf-β*(a reported repressor of Ptx3 transcription) decreased over time. In the conditioned medium of the 3D cocultures, TNF-α was not detected, while PTX3 and OPG levels were stable over time (79). Importantly, the expression of selected osteogenic (Bmp2, Alp, Spp1), and antioxidant (Nrf-2, Ho-1) genes were substantially affected by the applied 3D setting, indicating matrix-dependent effects on osteoblasts during an *S. aureus* infection (79).

In *S. aureus* osteomyelitis, mRNA and serum protein levels of G-CSF significantly increase in infected patients, contributing to bone loss through suppression of osteoblast function in favor of osteoclast formation (80), and to enhanced phagocytic activity of immune cells. G-CSF is also released directly by osteoblasts upon *S. aureus* infection, as well as GM-CSF and M-CSF. In fact, GM-CSF and G-CSF mRNA expression and protein secretion were substantially upregulated in cultured mouse and human osteoblasts following interaction with *S. aureus* (81). Furthermore, these cytokines were induced in unexposed osteoblasts separated from infected osteoblasts by means of a transwell system, thus pointing to a paracrine-autocrine regulatory mechanism. On the contrary, M-CSF secretion increased only in cultures of infected human osteoblasts (81). These CSFs allow osteoblasts to modulate the cellular composition of the bone microenvironment, favoring differentiation of myeloid progenitors towards osteoclasts and various innate immune cell fates (66).

### The osteoblast secretome: Chemokines

In response to *S. aureus* infection, osteoblasts also produce chemokines, members of the C-X-C-motif chemokine ligand (CXCL) and CC-motif (CCL) families, such as CCL2, CCL5, CCL7, CCL8, CCL10, CCL11, CCL13, CCL20, CCL26, and CXCL1, CXCL2, CXCL3, CXCL5, CXCL6 and CX3CL1 (57). Chemokines are traditionally known to act as immune cell chemoattractants, recruiting and activating components of the innate and adaptive immunity, however skeletal cells also are endowed with autocrine and paracrine chemokine signaling, which modulates bone turnover (82).

CCL3 and CXCL2 are known inflammatory mediators enhancing osteoclast formation and osteolysis (83). Their expression was documented in samples from osteolytic sites of patients with implant-associated infection (83). *In vitro* experiments showed that human primary osteoblasts released CCL3 and CXCL2 upon *S. aureus* infection (83). This indicates that, besides monocytes, also osteoblasts contribute to the sustained local production of these factors and the related enhanced RANKL-dependent bone resorption (82), in line with previous evidence in a mouse model (84) of *S. aureus*-induced osteomyelitis. Overexpression of CXCL2 would also result into inhibition of osteoblast formation through downmodulation of the ERK1/2 signaling upstream of RUNX2 (85). In accordance with a role of the CCL2-CCR2 axis in *S. aureus*-induced osteomyelitis, Ccr2-deficient mice had a higher bacterial load than wild type mice in a model of implant-associated *S. aureus* infection in which a bioluminescent bacterial strain was inoculated directly into the knee joint after implantation of an orthopedic-grade titanium pin. The infection was monitored *in vivo* by means of bioluminescence and *ex vivo* by colony-forming unit counting in the infected joint tissue (86). While the specific contribution of osteoblast-derived CCR2 could not be established in this model, the higher bacterial burden in Ccr2-deficient mice was ascribed to lower T cell and myeloid cell infiltration and overall reduced host defense against the pathogen in the absence of a functional CCR2/CCL2 axis.

*In vitro S. aureus* infection of human primary osteoblasts also causes a strong upregulation and release of CCL5 (also known as RANTES) compared to other cell types, such as endothelial and epithelial cells (87). The levels of this chemokine influence osteoclast and osteoblast formation and function, as highlighted in the CCL5-deficient mouse (88), besides acting as chemoattractant for monocytes/macrophages and T lymphocytes (89).

Additionally, in a mouse model of implant-associated osteomyelitis, CXCL10 and CXCL9 were up-regulated in the infected femurs versus controls at 3- and 14-days post-infection (90), suggesting a possible role of these chemokines (produced also by osteoblasts through TLR4 activation (91, 92)) in the pathological bone turnover occurring during Osteomyelitis. Of note, in clinical samples from *S. aureus*-infected patients and from a mouse model of MRSA skin infection, CXCL10 and CXCL9 have also been found to enhance the spontaneous release of the virulence factor SpA, however the underlying mechanism and the biological significance of this process are yet to be defined (93). In this regard, there is evidence that extracellular SpA contributes to biofilm formation by *S. aureus* (39), therefore the ability of certain chemokines to induce its release could paradoxically help the pathogen skip immune recognition *via* encasing in a protective layer of biofilm (94). A similar process could occur in principle in bone infections, further increasing the complexity of molecular interactions that underpin osteomyelitis.

Moreover, in a mouse model of endodontic infection-induced inflammation that mimics osteomyelitis of the jaw, the chemokines *Cxcl5*, *Cxcl2*, and *Cxcl13* were among the top upregulated genes in bone lesions (95).

Furthermore, osteoblasts express both CXCL12 (also known as SDF-1) and its receptor CXCR4. This signaling axis has been extensively studied in relation to the bone marrow niche (96) and implicated in skeletal homeostasis (97). Its involvement in bone remodeling during Osteomyelitis is quite likely, though not specifically investigated thus far, to the best of our knowledge.

Finally, the neutrophil chemoattractants CCL5, CXCL1, and CXCL8 and the chemokines related to T cell activation CXCL9, CXCL10, and CXCL11, were strongly upregulated in human-osteocyte-like cells in response to *S. aureus* invasion, in *ex vivo* infected human bone and in bone specimens from the infected acetabulum site of patients suffering from periprosthetic joint infection (48). CCL5 and CXCL10 proteins, but not CXCL8 were also confirmed to be secreted by infected osteocyte-like cells.

## Limitations of *in vitro* and *in vivo* models

Dissection of the mechanisms underlying bone infection is hindered by the involvement of a variety of cells within a complex, not easily accessible microenvironment. *In vivo* models have contributed significantly to our understanding of osteomyelitis and remain a valuable tool, even though they have inherent limitations (e.g., difficulties related to joint dimension and surgery procedures in small animals; different cortical bone composition and structure), as recently reviewed (98, 99).

Conventional *in vitro* 2D models and advanced microfluidics systems allow addressing specific hypotheses in a simplified environment (2, 100, 101), however, they also present some drawbacks: for instance, the effort to simplify the model may overlook molecules and/or cellular populations that are of pathogenetic relevance. Moreover, results achieved using cell lines may present inconsistencies with respect data obtained using primary cells. For example, *S. aureus* internalization has been shown to be 30-fold lower in human primary osteoblasts than in the human osteoblast cell line hFOB. On the other hand, human primary osteoblasts displayed significantly lower cell death and higher cytokine and chemokine production (87). These findings indicate that immortalized cell lines, though widely used, do not (always) faithfully reflect post-invasion and post-infection events occurring in primary cells and raise some doubts on the physiological relevance of cell line-based *in vitro* infection models. Conversely, the use of primary cells has drawbacks due to limited availability of material and inter-donor variability.

3D models that closely resemble the *in vivo* conditions have been implemented (79, 102, 103). In this regard, manufacturing of bioactive bone mimetic scaffolds that recapitulate texture and chemistry of the natural bone matrix provide unique experimental tools to study the interface between pathogens (including *S. aureus*) and both skeletal and immune cells in a tightly controlled setting. Yet, it is problematic to reproduce in 3D models the cellular and molecular complexity of the bone microenvironment.

Overall, consistency and translation of findings from both *in vivo* and *in vitro* settings is often problematic, and integration of different models and expertise is warranted.

## Conclusions and perspectives

In conclusion, our concise overview shows that osteoblasts are actively involved in the response to infection in *S. aureus* osteomyelitis. In this framework, they are engaged in complex osteoimmunological interactions. This implies the release of a variety of factors collectively described as the osteoblasts secretome, which, on one hand, recruit and activate immune cells, on the other modulate skeletal cell function. The major source of most inflammatory mediators in the bone marrow are professional innate and adaptive immune cells enrolled from the periphery or differentiated locally in the infected bone (8) (see **Figure 1**), as cell subsets specifically endowed with this function. Nevertheless, osteoblasts have turned out to be partners in defense (and crime) in *S. aureus* osteomyelitis through an arsenal of diverse factors (see **Figure 2**). Based on the established osteoblast-osteoclast bidirectional communication occurring in pathophysiological conditions (14), we would expect bone resorbing cells in turn impact on osteoblast function during infection. This aspect would be worth investigating, also considering the increasing heterogeneity recognized within the osteoclast lineage and its possible translational implications (24, 104).

The osteoblast secretome appears to be specific to the infectious agent, but whether any of its components or combinations of them can be used as specific biomarkers of the bone infection in the clinic remains to be evaluated. The possibility to manipulate the arsenal represented by the OB secretome for therapeutic purposes in addition to standard antibiotics treatments should also be considered. The double-faced nature of several cytokines and chemokines, which foster professional immune cells but sustain bone metabolism overbalance, makes this putative strategy challenging, though worth investigating, as prompted by an unmet medical need.

## Author contributions

GV and SC drafted the manuscript. GV and SC generated the figures. All authors contributed to the article and approved the submitted version.

## Funding

This work was partially supported by Fondazione Beppe e Nuccy Angiolini Onlus.

## Acknowledgments

GV and PV are supported by Fondazione Beppe e Nuccy Angiolini Onlus. We gratefully acknowledge their generous contribution to our research.

## Conflict of interest

BB receives royalties for reagents related to innate immunity and is inventor of patents related to PTX3 and other innate immunity molecules.

The remaining authors declare that the research was conducted in the absence of any commercial or financial relationships that could be construed as a potential conflict of interest.

## Publisher’s note

All claims expressed in this article are solely those of the authors and do not necessarily represent those of their affiliated organizations, or those of the publisher, the editors and the reviewers. Any product that may be evaluated in this article, or claim that may be made by its manufacturer, is not guaranteed or endorsed by the publisher.

## Glossary

**Table:**

IL-6	Interleukin 6
IL-8	Interleukin 8
IL-1β	Interleukin-1 beta
IL-12	Interleukin 12
VEGF	Vascular-Endothelial Growth Factor
CRP	C reactive protein
PRRs	Pattern-Recognition Receptors
TLRs	Toll-Like Receptors
PAMPs	Pathogen-Associated Molecular Patterns
LPS	Lipopolysaccharides
TLR2	Toll-like Receptor 2
TLR4	Toll-like Receptor 4
RANKL	Receptor Activator of Nuclear Factor κappa B Ligand
M-CSF	Macrophage Colony-Stimulating Factor
NOD	Nucleotide oligomerization domain
PMNs	Polymorphonuclear leukocytes
TNF-α	Tumor Necrosis Factor-alfa
CCL3	Chemokine (C-C motif) ligand 3
CXCL2	Chemokine (C-X-C motif) ligand 2
EFNB2	Membrane-bound ligand Ephrin B2
EPHB4	Ephrin receptor B4
FASL	Fas cell surface death receptor-ligand
FAS	Fas cell surface death receptor
SEMA3A	Semaphorin 3A
NRP1	Neuropilin-1
C-FMS	Colony-stimulating factor-1 receptor
RANK	Receptor Activator of Nuclear Factor κappa B
OPG	Osteoprotegerin
MSCRAMMs	Microbial Surface Components Recognizing Adhesive Matrix Molecules
FnBPA and B	Fibronectin-Binding Protein A and B
Cna	Collagen adhesin
SpA	Staphylococcus protein A
ClfA and B	clamping factor A and B
vWbp	von Willebrand factor-binding protein
SACs	Staphylococcal abscess communities
CCL5	Chemokine (C-C motif) ligand 5
MIP-1α	Macrophage Inflammatory Protein-1 alpha
MIP-1β	Macrophage Inflammatory Protein-1 beta
G-CSF	Granulocyte Colony-Stimulating Factor
MCP1	Monocyte Chemoattractant Protein-1
TSST-1	Toxic Shock Syndrome Toxin 1
NFATc1	Nuclear Factor Of Activated T Cells 1
TNFR1	Tumor Necrosis Factor Receptor 1
RUNX2	Runt-related transcription factor 2
COL1a1	Collagen type I alpha 1 chain
ALP	Alkaline phosphates
FAK	Focal Adhesion Kinase
EGFR	Epidermal Growth Factor Receptor
c-Src	Proto-oncogene
cellular Src	
MRSA	Methicillin-Resistant Staphylococcus aureus
IFC	imaging flow cytometry
TRAIL	Tumor Necrosis Factor-Related Apoptosis-Inducing Ligand
WNT5A	WNT family member 5A
WNT16	WNT family member 16
GM-CSF	Granulocyte-Macrophage Colony-Stimulating Factor
IGF	insulin growth factor
IGFBP	insulin growth factor-binding protein
CXCL12	Chemokine (C-X-C motif) ligand 12
SCF	Stem cell factor
IL-7	Interleukin 7
NF-kB	Nuclear factor kappa-light-chain-enhancer of activated B cells
JNK	c-Jun N-terminal kinase
IL-33	Interleukin 33
IL-32	Interleukin 32
IL-1α	Interleukin 1 alfa
IL-24	Interleukin 24
TNFSF14	Tumor Necrosis Factor Superfamily Member 14
Th17	T helper 17
IL-17	Interleukin 17
COX2	Cyclooxygenase 2
PGE2	Prostaglandin E2
Th1	T helper 1
TGFβ1	Transforming Growth Factor beta 1
IL-18	Interleukin 18
IL-18BP	Interleukin 18 Binding Protein
eNOS	Endothelial Nitric Oxide Synthase
IL-27	Interleukin 27
PTX3	Pentraxin 3
Bmp2	Bone morphogenetic protein 2
Spp1	Secreted Phosphoprotein 1
Nrf-2	Nuclear factor erythroid 2-related factor 2
Ho-1	heme oxygenase-1
CCL2	Chemokine (C-C motif) ligand 2
CCL7	Chemokine (C-C motif) ligand 7
CCL8	Chemokine (C-C motif) ligand 8
CCL10	Chemokine (C-C motif) ligand 10
CCL11	Chemokine (C-C motif) ligand 11
CCL13	Chemokine (C-C motif) ligand 13
CCL20	Chemokine (C-C motif) ligand 20
CCL26	Chemokine (C-C motif) ligand 26
CXCL1	Chemokine (C-X-C motif) ligand 1
CXCL2	Chemokine (C-X-C motif) ligand 2
CXCL3	Chemokine (C-X-C motif) ligand 3
CXCL5	Chemokine (C-X-C motif) ligand 5
CXCL6	Chemokine (C-X-C motif) ligand 6
CX3CL1	chemokine (C-X3-C Motif) ligand 1
ERK1/2	Extracellular signal-regulated kinase 1/2
CCR2	C-C chemokine receptor type 2
CXCL10	Chemokine (C-X-C motif) ligand 10
CXCL9	Chemokine (C-X-C motif) ligand 9
CXCL13	Chemokine (C-X-C motif) ligand 13
CXCR4	C-X-C chemokine receptor type 4
CXCL11	Chemokine (C-X-C motif) ligand 11
hFOB	Human fetal osteoblast
